# Prediction of Pathogenic Factors in Dysbiotic Gut Microbiomes of Colorectal Cancer Patients Using Reverse Microbiomics

**DOI:** 10.3389/fonc.2022.882874

**Published:** 2022-04-27

**Authors:** Haihe Wang, Kaibo Zhang, Lin Wu, Qian Qin, Yongqun He

**Affiliations:** ^1^ Department of Immunology and Pathogen Biology, Lishui University, Lishui, China; ^2^ Center of Computer Experiment, Lishui University, Lishui, China; ^3^ Unit for Laboratory Animal Medicine, University of Michigan, Ann Arbor, MI, United States; ^4^ Department of Microbiology and Immunology, University of Michigan Medical School, Ann Arbor, MI, United States; ^5^ Center of Computational Medicine and Bioinformatics, University of Michigan, Ann Arbor, MI, United States; ^6^ Comprehensive Cancer Center, University of Michigan Medical School, Ann Arbor, MI, United States

**Keywords:** reverse microbiomics, ontology, gut microbiome, colorectal cancer, bioinformatics, reverse vaccinology

## Abstract

**Background:**

Gut microbiome plays a crucial role in the formation and progression of colorectal cancer (CRC). To better identify the underlying gene-level pathogenic mechanisms of microbiome-associated CRC, we applied our newly developed Reverse Microbiomics (RM) to predict potential pathogenic factors using the data of microbiomes in CRC patients.

**Results:**

Our literature search first identified 40 bacterial species enriched and 23 species depleted in the guts of CRC patients. These bacteria were systematically modeled and analyzed using the NCBI Taxonomy ontology. Ten species, including 6 enriched species (e.g., *Bacteroides fragilis*, *Fusobacterium nucleatum* and *Streptococcus equinus*) and 4 depleted species (e.g., *Bacteroides uniformis* and *Streptococcus thermophilus*) were chosen for follow-up comparative genomics analysis. Vaxign was used to comparatively analyze 47 genome sequences of these ten species. In total 18 autoantigens were predicted to contribute to CRC formation, six of which were reported with experimental evidence to be correlated with drug resistance and/or cell invasiveness of CRC. Interestingly, four human homology proteins (EDK89078.1, EDK87700.1, EDK89777.1, and EDK89145.1) are conserved among all enriched strains. Furthermore, we predicted 76 potential virulence factors without homology to human proteins, including two riboflavin synthase proteins, three ATP-binding cassettes (ABC) transporter protein family proteins, and 12 outer membrane proteins (OMPs). Riboflavin synthase is present in all the enriched strains but not in depleted species. The critical role of riboflavin synthase in CRC development was further identified from its hub role in our STRING-based protein−protein interaction (PPI) network analysis and from the finding of the riboflavin metabolism as the most significantly enriched pathway in our KEGG pathway analysis. A novel model of the CRC pathogenesis involving riboflavin synthase and other related proteins including TpiA and GrxC was further proposed.

**Conclusions:**

The RM strategy was used to predict 18 autoantigens and 76 potential virulence factors from CRC-associated microbiome data. In addition to many of these autoantigens and virulence factors experimentally verified as reported in the literature, our study predicted many new pathogenetic factors and developed a new model of CRC pathogenesis involving the riboflavin synthase from the enriched colorectal bacteria and other associated proteins.

## Introduction

Colorectal cancer (CRC) is a heterogeneous disease of the intestinal epithelium that is characterized by the accumulation of mutations and dysregulated immune response. Up to 90% of the disease risk is due to various genetic and environmental factors including age, genetics, lifestyle, diet, inflammation, immune regulation, metabolism and genotoxin production (1, 2). Accumulating evidences suggest that human gut microbiome plays a key role in the incidence and progression of CRC (3). Significant differences have been found in intestinal microbial communities between CRC patients and healthy individuals, and between tumor and adjacent mucosa (4–6). Chronic inflammatory diseases of the intestinal tract, such as inflammatory bowel disease (IBD), also increase the risk for the development of CRC (7). Microbial dysbiosis frequently occurs as a consequence of mucosal inflammation (8). Experiments in mouse models suggest that a dysbiotic gut microbiota can instigate or worsen gut inflammation in some instances (9). Dysbiosis is partly characterized by the expansion of bacterial taxa, such as *Fusobacterium nucleatum* (10, 11), *Enterococcus faecalis*, *Bacteroides fragilis* (12), *and Escherichia coli*, which were found to be enriched in the feces of CRC patients and promote the occurrence of CRC (4). *B. fragilis* is the most frequent anaerobe isolated in clinical cases of diarrhea, peritonitis, and has been epidemiologically linked to CRC (12, 13). *F. nucleatum* could induce the expression/secretion of proinflammatory cytokines IL17A and TNF in colorectal tumors (14–16). Studies showed, *F. nucleatum* derived factors such as Fap2 is able to regulate tumor-immune evasion and inhibit NK cell killing of various tumors (17). Despite enormous efforts and substantial progress have been made to understand the composition and role of the human intestinal microbiota in CRC, many functional aspects remain unresolved.

Metagenomic sequencing is emerging as a powerful approach to microbiome investigation. Up to now, there are several strategies implemented for the analysis of microbial communities, such as shotgun metagenomics, 16S rDNA sequencing, metagenome-wide association study (MGWAS) (18) and genome-wide association studies (GWAS) (19). Metagenomics strategies provide quick information about the taxonomic composition of microbial communities. Most current microbiota and dysbiosis studies end with the identification of bacteria at different taxonomical levels (e.g., species, genus, and family) enriched or depleted in patients compared to healthy controls. However, it has become a big challenge to further study the underlying gene-based molecular pathogenic mechanisms.

**Figure 1:**
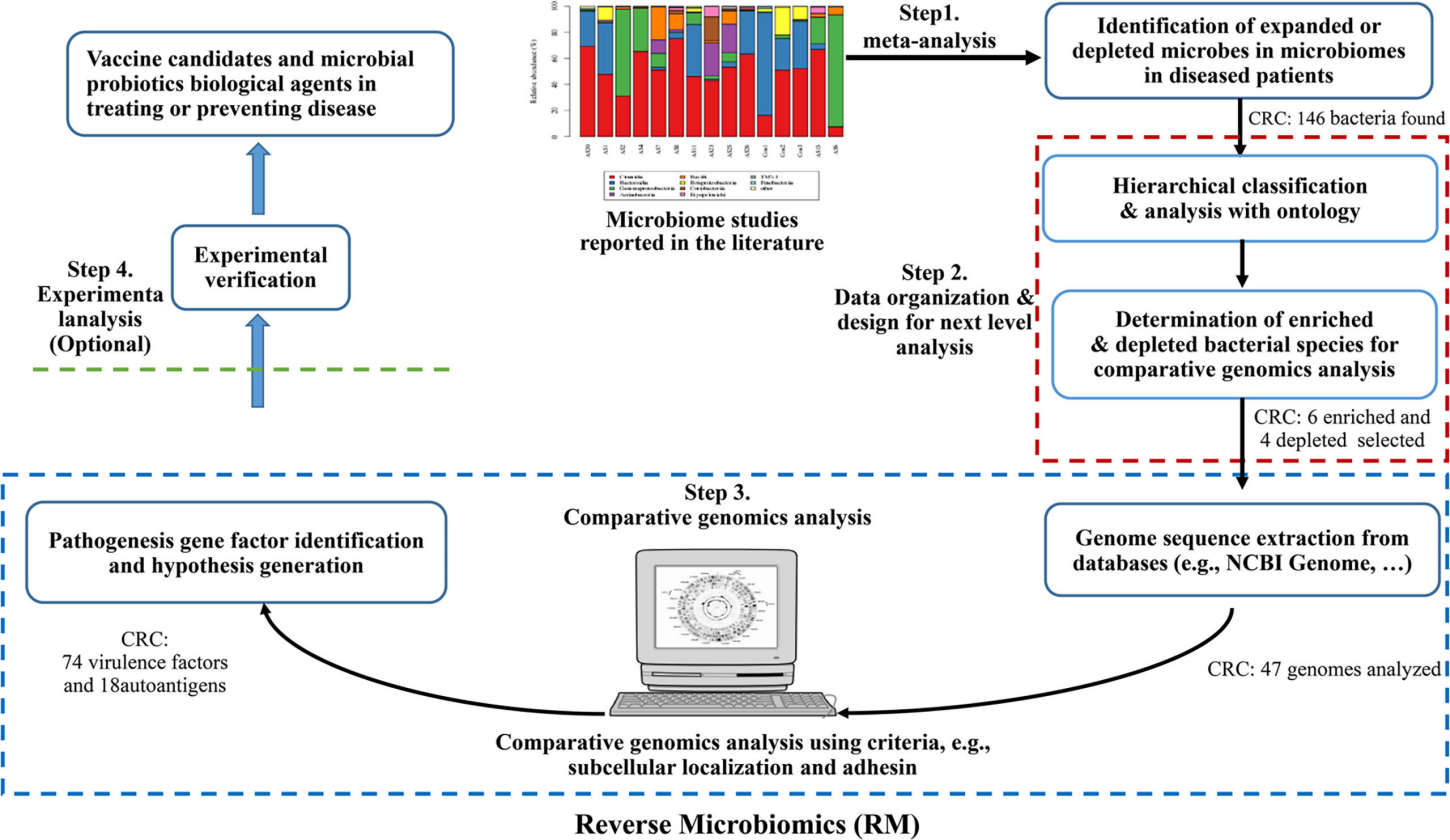
The Reverse Microbiomics workflow and its application for CRC. The RM strategy includes four steps: (i) identification of differentially regulated species by literature mining and meta-analysis from published experimental studies; (ii) ontology-based hierarchical analysis of these species; (iii) comparative genomics analysis of autoantigens and virulence factors contributing to microbiome-associated diseases, and (iv) New hypotheses may also be generated and experimentally verified.

The Reverse Microbiomics (RM) is a new strategy we recently developed to address the challenge of identifying the molecular pathogenic mechanisms or potential vaccine antigen candidates against microbiome-related diseases (20). The RM strategy includes four steps: (i) identification of differentially regulated species by literature mining and meta-analysis; (ii) ontology-based hierarchical analysis of these species; (iii) comparative genomics analysis of autoantigens and virulence factors contributing to microbiome-associated diseases, leading to new hypothesis generation, and (iv) selective experimental verification (optional) (**Figure 1**). Our research first collected all the related differentially regulated species by meta-analysis. Ontology is a human-and computer-interpretable representation and classification of the types, properties, and relations of entities in a particular domain (21). Ontologies support semantic reasoning, and enable people and machines to make logical inferences. In our RM study, we used ontology to classify all identified bacteria and identify specific species for further analysis. Inspired by reverse vaccinology (RV) (22), our RM comparative genomics analysis focuses on prediction of specific criteria such as subcellular localization and adhesin. We have applied RM to study rheumatoid arthritis (RA) and predicted many autoantigens or virulence factors contributing to RA. Our study also generated many interesting hypotheses that deserve further experimental verification (20).

In this study, we report our further development and application of the RM strategy for systematic prediction of pathogenic factors (including autoantigens and virulence factors) that likely contribute to the CRC pathogenicity.

## Methods

### Meta-Analysis of CRC-Related Gut Microbiome Profiles

As the first RM step (**Figure 1**), the CRC related microbiota study data were collected from PubMed using the keywords of “microbiome”, “microbiota”, and “colorectal cancer”. The enriched or depleted bacteria to be included in the study required that the bacteria must come from experiment-based research papers. Experimental assays, including traditional culture-based approaches, 16S rRNA sequencing and metagenomic sequencing, were identified in our study. The taxonomy ID, rank, location, PMID, and description information of differentially changed bacteria were collected in an Excel file.

### Ontological Classification of CRC-Related Gut Microbiome Profiles

All the enriched or depleted bacteria identified from the meta-analysis described above were mapped to corresponding terms in the NCBI Taxonomy ontology (NCBITaxon, https://obofoundry.org/ontology/ncbitaxon.html), which is an ontology that represents all the organisms stored in the NCBI Taxonomy database (23) and the taxonomic relations among these organisms. Using the enriched or depleted bacteria as input, the Ontofox (24) was applied to extract bacterial terms and their associated ancestors in NCBITaxon in a hierarchical structure (http://ontofox.hegroup.org/). Such Ontofox output was visualized using Protégé OWL-editor (25), downloaded from https://protege.stanford.edu/. Screenshots were also made from the Protégé displays. The Ontofox output was also incorporated into the Ontology of Host-Microbiome Interactions (OHMI) (26), and new semantic axioms were also added in OHMI to represent the relations among these bacteria and CRC. While the OHMI representation is not required in our RM pipeline, such an ontology representation allows the information semantically stored and available for future computer-assisted reasoning (26).

### Comparative Genomics Analysis Using Vaxign

For more specific comparative genomic analysis, we preferably selected those pairs of bacterial species that include both enriched and depleted bacteria within the same genus, with the aim to find targeted genes present in the enriched bacteria but absent from the depleted bacteria. To enhance our findings, we chose multiple pairs of such bacterial species. After the bacterial species were decided, the NCBI BioProject database was used to obtain all the protein sequences of the representative strains in these species. To standardize the bioinformatics analysis, related information including strain names, NCBI BioProject, NCBI BioSample numbers, protein size numbers were first summarized in Excel. The NCBI BioProject numbers were used by the Vaxign dynamic analysis to automatically retrieve proteomes that contains all protein sequences. Vaxign automatically retrieved the protein sequences of the chromosome and any possible plasmid of these bacteria. For each protein, Vaxign was used to analyze various features including subcellular localization, conservation among different strains, exclusion from depleted strains, and homology with human proteins (27, 28). The adhesin probability cutoff of 0.51 was used for adhesin prediction. The Vaxign-ML machine learning tool is available at http://www.violinet.org/vaxign2/vaxign-ml.

### Antigenicity Prediction

Antigenicity scores of potential pathogenic antigens were calculated by the VaxiJen v2.0 server (http://www.ddg-pharmfac.net/vaxijen/VaxiJen/VaxiJen.html). This software uses the z-descriptor composed of multiple physicochemical properties of proteins to predict their antigenicity from FASTA-submitted amino acid sequences using partial least squares discriminant analysis (DA-PLS). The antigens having values more than 0.4 were considered potentially antigenic as described by Doytchinova and Flower (29).

### PPI Network Construction and KEGG Pathway Enrichment Analysis

The PPI network of 71 proteins (58 no human homology proteins and 13 human homology proteins) from *F. nucleatum* were identified using the Search Tool for the Retrieval of Interacting Genes/Proteins (STRING) database (string−db.org; release 11.0) (30). The minimum required interactions with confidence scores of ≥0.4 were selected as significant and the most number of nodes were visualized by Cytoscape v3.6.1 (31). The hub proteins in the PPI network were selected by the cytoHubba plug−in, according to node degree ≥10 (32).The functional and pathway enrichment analysis of hub proteins was performed by KOBAS 3.0 (http://kobas.cbi.pku.edu.cn/).

## Results

### Bacterial Profiles Associated With CRC in Gut Microbiomes

**Figure 2:**
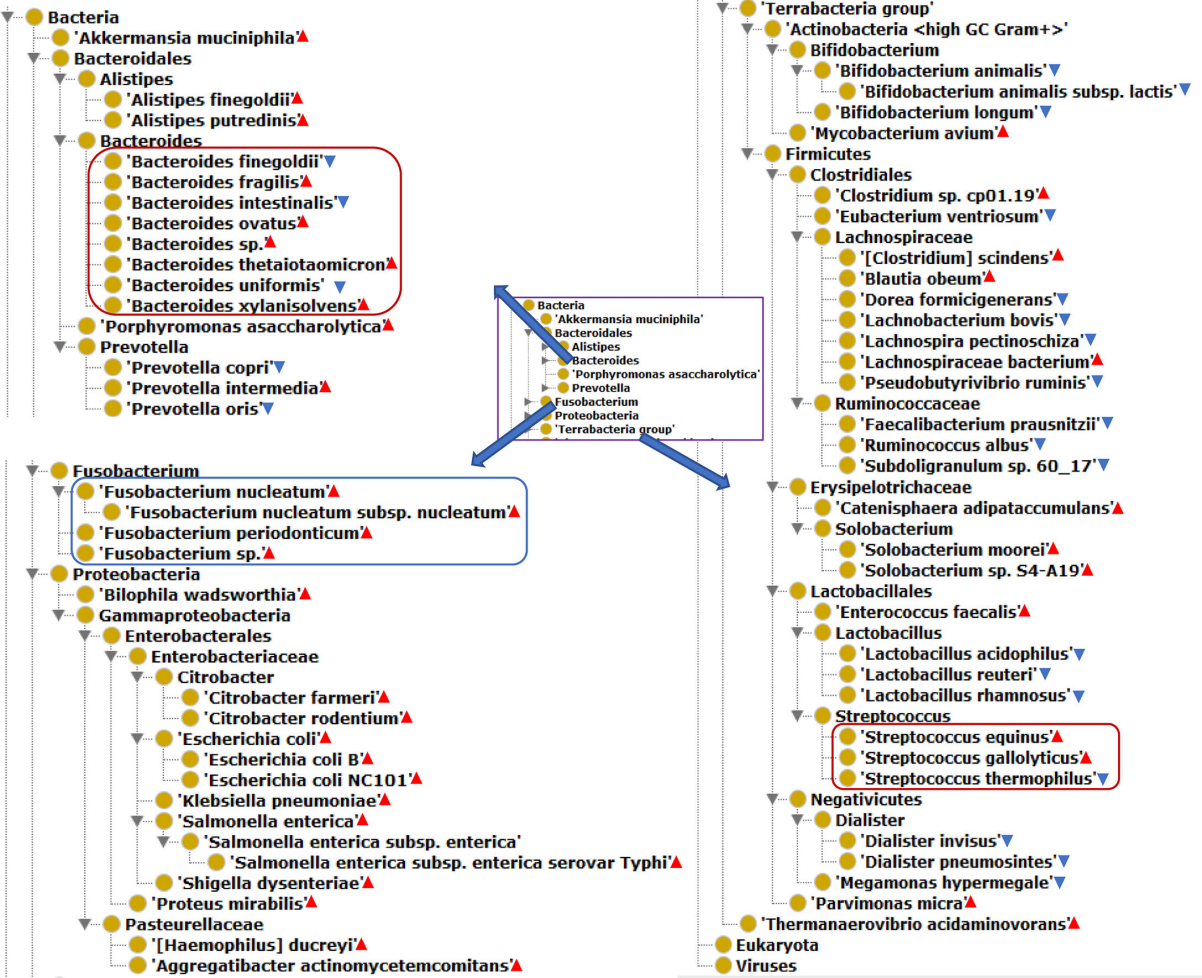
The hierarchical structure of 63 significantly changed bacteria from the guts of CRC patients. The bacterial species presence in the gut of CRC patients. Color meanings: Red triangle - enriched species; Blue triangle - depleted species.

Our literature mining and annotation of 79 peer-reviewed publications found 146 bacteria statistically differentially enriched or depleted in the gut of CRC patients compared with healthy (**Supplementary Tables 1**, **2**). Out of the 146 identified bacteria, 63 were at the levels of species, of which 40 were enriched and 23 depleted species. **Figure 2** displays these 63 bacterial species in an ontology-based hierarchical structure. In the genus of *Bacteroides*, five species (including *B. fragilis*, *B. ovatus*, *Bacteroides* sp., *B. thetaiotaomicron*, and *B. xylanisolvens*) were enriched, and three species (including *B. uniformis*, *B. intestinalis*, and *B. finegoldii*) were depleted. In the genus of *Streptococcus*, two species including *S. equinus* and *S. gallolyticus* were enriched, and one species of *S. thermophilus* was depleted. In the genus of *lachnospiraceae*, four species were depleted, and three species were enriched in the gut of CRC patients. *F. nucleatum* was found to be the most prevalent in CRC patients, and it played critical roles in promoting proinflammatory response and neoplastic development (33).

### CRC-Related Microbial Genes by RM Analysis

**Figure 3:**
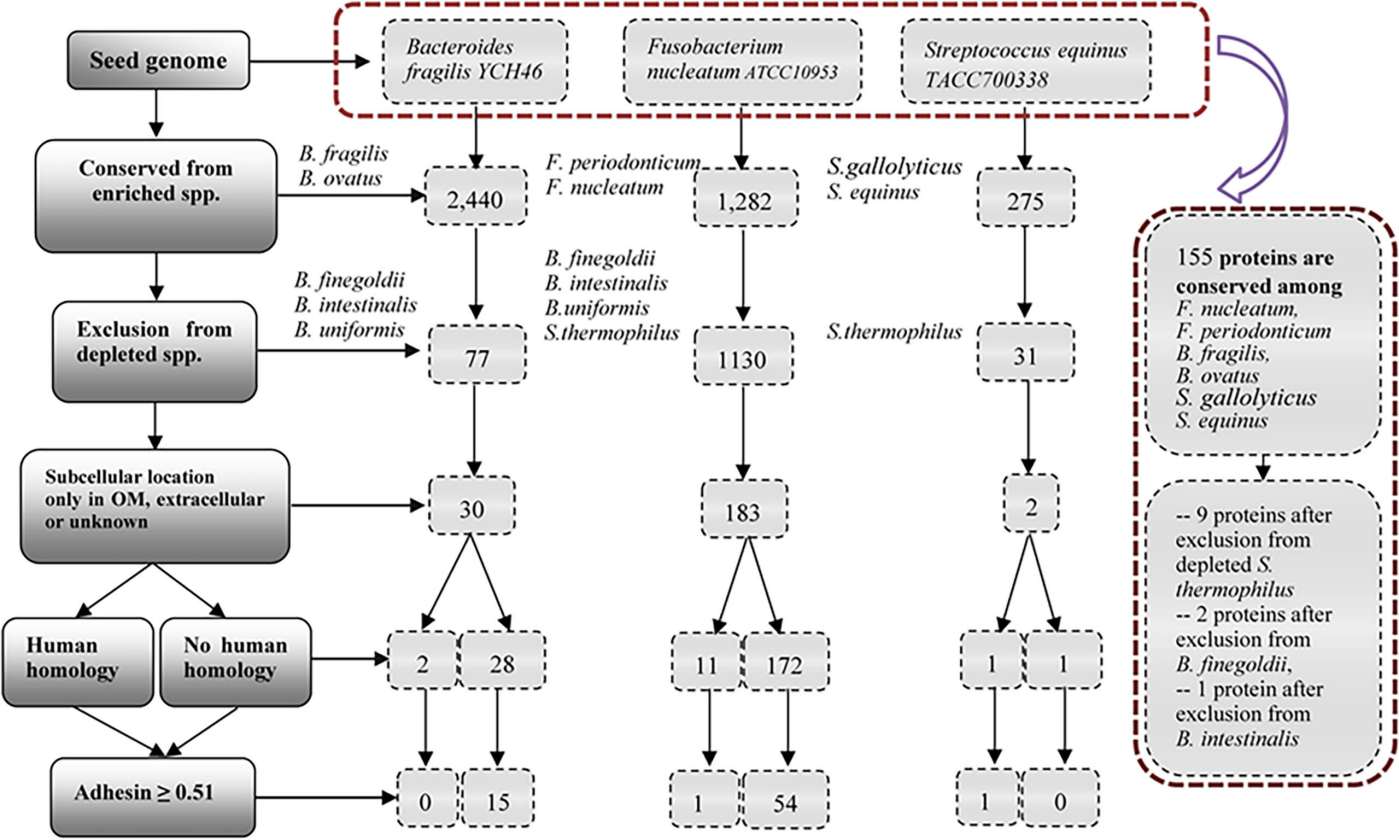
Vaxign analysis overall workflow and related results. The overall project workflow and related results were shown in **Figure 3**. In the group of *Bacteroides*, *B. fragilis YCH46* was used as seed genome. RM predicted 30 proteins, including two proteins with human homology and 28 proteins without human homology. In the group of *Streptococcus*, *S. equinus TACC700338* was used as seed genome, and predicted one human homology proteins, and one proteins without human homology. In the group of *Fusobacterium*, *F. nucleatum ATCC10953* was used as seed genome, and compared with the increased and decreased genomes of the group of *Bacteroides* and *Streptococcus*. 54 proteins without human homology and 11 human homology proteins were identified. Finally, *F. nucleatum ATCC10953* as seed genome, compared with 25 enriched genomes of *F.nucleatum*, *F. periodonticum*, *B. fragilis*, *B.ovatus*, *S.gallolyticus*, and *S. equinus*, and 155 proteins were conserved among all the enriched species. Exclusion from depleted genomes of *S. thermophilus*, *B. finegoldii* and *B. intestinalis*, we obtained nine proteins, two proteins and one proteins, respectively.

We hypothesized that pathogenic virulence factors are most likely expressed in the enriched species and do not exist in depleted species in microbiome-associated diseases. To demonstrate the usage of the RM strategy, we focused on ten bacterial speices which have been well known to play an important role in the pathogenesis of CRC (**Figure 3**). We collected and annotated 47 genome sequences from NCBI database, and analyzed them using Vaxign (**Supplementary Table 3**).

**Figure 3** provides the overall project workflow and related results. In the enriched group of *Bacteroides*, *B. fragilis* strain YCH46 was used as seed genome. Among the 4,278 proteins, 2,440 proteins were conserved among five enriched genomes of *B. fragilis* and *B. ovatus*, and 77 proteins were predicted to be absent from the 11 depleted genomes of *B. finegoldii*, *B. intestinalis*, and *B. uniformis*. By including only those proteins located in the outer membrane, extracellular or unknown locations, we obtained 30 proteins, including two proteins with human homology and 28 proteins without human homology. Adhesins are important for pathogens to invade host cells. Our Vaxign analysis also predicted 15 no-human-homology proteins being possible adhesins.

In the enriched group of *Streptococcus*, *S. equinus* strain TACC700338 was used as seed genome. Among the 1,862 proteins in the genome, 275 proteins are conserved among the nine increasing genomes of *S. gallolyticus* and *S. equinus*, and 31 proteins were predicted to be absent from the 11 decreasing genomes of *S. thermophilus.* Furthermore, two proteins, including one human homology protein, and one no-human-homology protein were found to locate in the outer membrane, extracellular or unknown locations.

The other enriched bacterial species studied in this work are *Fusobacterium nucleatum* and *F. periodonticum* (**Figure 2**). *Fusobacterium* is the most prevalent bacterial genus found in the dysbiotic gut microbiome of CRC patients (14, 15). However, our literature survey did not find any depleted species in the genus of *Fusobacterium* in the colon microbiomes of CRC patients. To enhance our identification of virulence factors in *F. nucleatum*, we used the genome of *F. nucleatum* strain ATCC10953 as the seed genome, and the genomes of depleted bacteria of *Bacteroides* and *Streptococcus* as negative controls (**Figure 3**). Specifically, 1,130 of these proteins were found to be absent from 22 genomes of depleted *B. finegoldii*, *B. intestinalis, B.uniformis*, and *S. thermophilus*. A total of 183 proteins, including 11 proteins with human homology and 172 proteins without human homology were found to locate in the outer membrane, extracellular or unknown locations. Among these 172 proteins, 54 no-human-homology proteins and one human-homology proteins were predicted to be adhesins.

Finally, we used *F. nucleatum* strain ATCC10953 as seed genome and compared it with the 25 enriched genomes of *F.nucleatum*, *F. periodonticum*, *B. fragilis*, *B.ovatus*, *S.gallolyticus*, and *S. equinus*. A total of 155 proteins were found to be conserved among all the enriched species. From these 155 proteins, we further found nine proteins, two proteins and one protein after exclusion from depleted genomes of *S. thermophilus*, *B. finegoldii* and *B. intestinalis*, respectively. Two of these 12 proteins belong to the same protein. The remaining 11 proteins include seven no-human-homology proteins and four human-homology proteins.

### Prediction and Evaluation of 18 Autoantigens for CRC Pathogenicity

Our RM study found 18 bacterial proteins having homology with human proteins. Two of these proteins come from enriched strains *B. fragilis* and *B. ovatus*, 15 proteins from enriched *F. periodonticum* and *F. nucleatum*, and one protein from enriched *S. gallolyticus* and *S. equinus* (**Table 1** and **Supplementary Table 4** **)**. Most interestingly, four human homology proteins (EDK89078.1, EDK87700.1, EDK89777.1, and EDK89145.1) are conserved among all these six enriched strains.

**Table 1:** Vaxign predicted gut microbiome human homology proteins that likely become autoantigens in human diseases.

RefSeq	Adhesin	Protein Name	Disease* and PubMed refs
***B. fragilis* proteins**			
BAD48998.1	0.187	putative oxidoreductase	CRC (31436301)
BAD46985.1	0.327	conserved hypothetical protein	
***S. equinus* proteins**			
EFM27067.1	0.555	glutathione peroxidase	CRC (23977205)
***F. nucleatum* proteins**			
EDK89817.1	0.102	Snf2 family helicase	CRC (8415573; 26398009; 24870791)
EDK89840.1	0.168	branched chain amino acid ABC superfamily ATP binding cassette transporter ABC protein	CRC(25689483; 29789423)
EDK89078.1	0.220	metal ion ABC superfamily ATP binding cassette transporter ABC protein	CRC (29789423, 25689483, 26043974, 31810909)
EDK87700.1	0.337	glutathione peroxidase	CRC (23977205)
EDK89306.1	0.216	hypothetical protein FNP_1525	
EDK88909.1	0.444	aldose 1-epimerase	
EDK88743.1	0.366	possible endodeoxyribonuclease	
EDK88512.1	0.568	NADH dehydrogenase	
EDK89426.1	0.096	tetratricopeptide repeat family protein	
EDK88907.1	0.381	possible outer membrane protein/esterase	
EDK88810.1	0.121	dCMP deaminase	
EDK88593.1	0.386	3-hydroxybutyryl-CoA dehydrogenase	
EDK88584.1	0.297	M16B family zinc (Zn2+) peptidase	
EDK89777.1	0.154	triose-phosphate isomerase	
EDK89145.1	0.327	phosphopantothenate–cysteine ligase	

*Disease here means that disease where the bacterial’s homologous human protein is confirmed to be an autoantigen for the disease. The PubMed paper reference is also provided. The detailed information can be found in **Supplementary Table 4**.

To evaluate our RM predictions, we compared these results with existing literature data. Among the 18 human homology proteins, six autoantigens were verified based on literature study to get involved in CRC, and the other 12 autoantigens not reported in the literature to participate in CRC pathogenesis (**Table 1** and **Supplementary Table 4**). Based on the results, we can hypothesize that these 12 human homology proteins likely trigger autoimmunity and stimulate autoimmune inflammatory responses and histomorphological damage in CRC patients.

### Identification and Evaluation of 76 Adhesin Proteins as Potential Virulence Factors of CRC

A total of 76 adhesin proteins with no human homology were identified and predicted to be associated with CRC pathogenesis. These proteins included four AT family autotransporter proteins, three ATP binding proteins, two riboflavin synthase proteins, 11 outer membrane proteins (OMPs), 39 hypothetical proteins, and 17 other proteins (**Table 2** and **Supplementary Table 5**). In these proteins, 12 proteins had antigenicity score over 0.7, and EDK88281.1 had the highest antigenicity score of 1.0519, suggesting that these proteins likely to elicit a strong immune response and induce inflammatory reaction in CRC. Meanwhile, seven proteins (i.e.,EDK89571.1, EDK89346.1, EDK89344.1, EDK89343.1, EDK88974.1, EDK88640.1, EDK88425.1) are conserved among all the enriched species, and they are absent from 11 decreased species genomes of *S. thermophilus*.

**Table 2:** Vaxign predicted no human homology proteins as potential CRC virulence factors.

Protein Name	Localization
***F. nucleatum* proteins**	
AT family autotransporter protein (4)	OM (2); UN (2)
ATP binding protein (3)	OM (1); UN (2)
riboflavin synthase (2)	CY (2)
outer membrane protein (7)	OM (3); UN (4)
hypothetical protein (32)	OM (5); UN (26); CY (1)
Other protein (13)	OM (3); UN (6); CY (4)
***B. fragilis* proteins**	
outer membrane protein (4)	OM (4)
hypothetical protein (7)	EX (1); UN (6)
Other protein (4)	OM (2); EX (1); UN (1)

EX, Extracellular; UN, Unknown; OM, Outer Membrane; CY, Cytoplasmic.

### The Key Hub Proteins and Pathways

**Figure 4:**
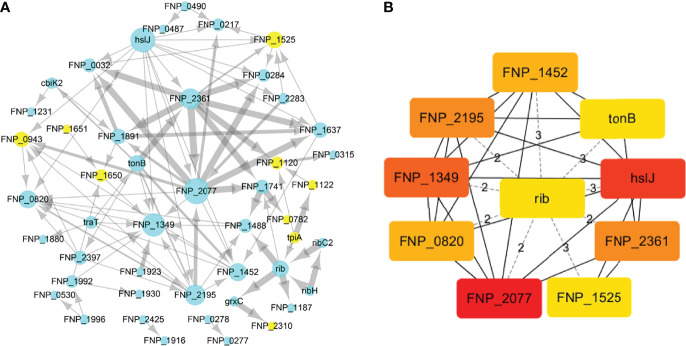
The PPI network construction and hub proteins analysis of 71 proteins. **(A)** The PPI network, mint green nodes represent the no human homology proteins, while yellow nodes represent human homology proteins, the node size indicates the node degree. **(B)** Top ten hub proteins in PPI network, the hub proteins were selected by the cytoHubba plug−in, according to node degree ≥10, top-ranked nodes are shown with a color scheme from highly essential (red) to essential (green). Rib: riboflavin synthase; HslJ: possible heat shock protein; TonB: ligand gated channel protein TonB; FNP_2077 and FNP_2361: AT family autotransporter; FNP_1349: hypothetical protein; FNP_2195: hypothetical protein; FNP_1452: iron (Fe3+) ABC superfamily ATP binding cassette transporter binding protein; FNP_1525: hypothetical protein FNP_1525; FNP_0820: possible outer membrane protein P1.

**Figure 5:**
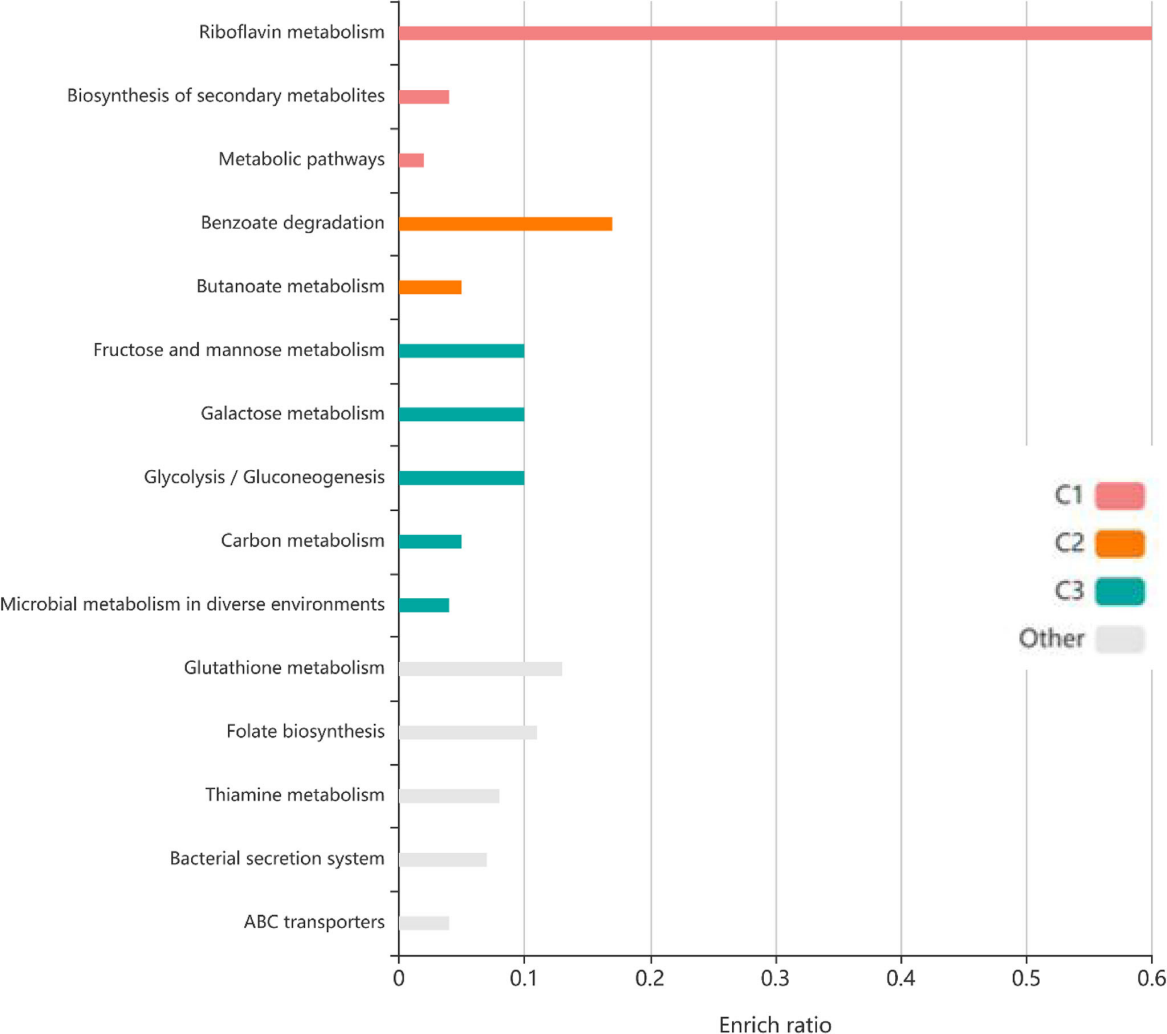
The KEGG pathway enrichment analyses of 71 protein. Top ten KEGG pathways of proteins, each row represents an enriched function, and the length of the bar represents the enrich ratio. The color of the bar represents different clusters.

To identify the interactions between proteins, the PPI network of the 71 proteins from *F. nucleatum* was constructed using STRING. The PPI network consisted of 76 nodes and 112 edges with a confidence score of ≥ 0.4 (**Figure 4A**). A total of 10 key hub proteins were selected from the PPI network with a degree of ≥ 10 (**Figure 4B**), including EDK89344.1 (Rib), EDK89837.1 (FNP_2077), EDK88710.1 (HslJ), EDK89132.1 (FNP_1349), EDK88281.1 (TonB), EDK89234.1 (FNP_1452), EDK87587.1 (FNP_2195), EDK88621.1 (FNP_0820), EDK89306.1 (FNP_1525) and EDK87750.1 (FNP_2361). KEGG analysis indicated that these proteins are primarily involved in riboflavin metabolism (ranked as the top 1 pathway in our analysis), biosynthesis of secondary metabolites, metabolic pathways, and benzoate degradation (**Figure 5**). The PPI network and KEGG analysis suggested that riboflavin and riboflavin metabolism pathway likely involved in CRC pathogenesis.

## Discussion

Three major contributions have been made in this study. First, using an ontology-based literature mining method, we found 63 valuable bacterial species that were up- or down-regulated in the gut microbiomes from the CRC patients. Second, using the Reverse Microbiomics (RM) strategy, we predicted 18 autoantigens and 76 potential virulence factors associated with CRC. Third, our further analysis identified the important role of riboflavin synthase in CRC pathogenesis, as demonstrated by its presence in all enriched bacterial species and absence in all depleted species in gut microbiomes of CRC, and its associated genes in the potential CRC pathogenesis network and pathway analyses.

The RM strategy is the first methodology for predicting potential virulence factors using reverse vaccinology based bioinformatics analysis (20). The major strength of the RM approach is its application in addressing the challenge of predicting the fundamental gene-level molecular mechanisms of microbiome-related diseases. The 16S rDNA and shotgun metagenomics technologies have been powerful to identify bacteria in disease-associated microbiomes. More existing disease microbiome studies ended with the identification of bacteria in taxonomical level (e.g., species and genus) without defining or predicting molecular mechanisms. Several network-based reverse ecology approaches such as NetCooperate (34), RevEcoR (35), PopCOGenT (36) have also been developed to study the interfaces between species and their environments, and predict the interactions between species. However, underlying gene-level molecular mechanisms of microbiome-related diseases remain poorly defined. To address this challenge, the RM strategy offers a novel and effective approach to identify and predict disease-relevant pathogenic virulence factors or self-antigens associated with microbiome-associated diseases.

The 18 human homology proteins identified in our study include six proteins known to be CRC-related human autoantigens and 12 newly identified proteins. These six known autoantigens that have homology with four human autoantigens experimentally verified to involve a pathogenic role in CRC: ATP-binding cassettes (ABC) transporter protein family proteins (37), TATA-binding protein (38–40), SDR family protein (41), and glutathione peroxidase protein (42) (**Table 1** and **Supplementary Table 4**). For example, EDK89078.1 and EDK89840.1 have homology with ABC transporter protein family proteins. In humans, ABC transporter proteins play clinically important roles in drug metabolism and resistance (37, 43). Studies showed that ATP-binding cassette protein mediates 5-fluorouracil resistance (44) and promotes tumor cell invasiveness in CRC (45). The other examples include EFM27067.1 and EDK87700.1, which have homology with human glutathione peroxidase protein. The glutathione peroxidase-2 (GPx2) have a dual role in carcinogenesis of CRC (42). The identification of these known autoantigens in CRC pathogenesis identified in our study confirmed the validity of our RM prediction.

Meanwhile, we found 12 new autoantigens (**Table 1** and **Supplementary Table 4**) that are likely to induce autoimmunity and contribute to CRC formation. For example, EDK89777.1 has homology with human triosephosphate isomerase (TPI) 1 which is one of the most important hallmarks for fast-growing tumor cells. Studies found that TPI was overexpressed in human gastric cancer (GC) and CRC tissues relative to the normal tissue (46, 47). EDK89426.1 has homology with human tetratricopeptide repeat (TPR) protein 28. Studies revealed that p58 (a member of TPR family) can inhibit the activity of interferon-induced RNA-dependent protein kinase (PKR). p58-overexpressing cells exhibited a transformed phenotype, growing at faster rates and higher saturation. An inoculation of nude mice with p58-overexpressing cells gave rise to the production of tumors (48). The roles of TPI, TPR, and the others of the 12 newly identified proteins in CRC formation are worth further investigation.

Our study also identified 76 no-human-homology proteins as potential pathogenic factors of CRC (**Table 2** and **Supplemental Table 5**). Among these proteins, many outer membrane proteins (OMPs), ABC transporter proteins have been reported to be association with CRC, and the other proteins are predicted to be new pathogenic factors. For example, Fap2 is an important OMP encoded by the *Fap2* gene of *F. nucleatum* that participates in the binding of *F. nucleatum* to cancer cells and to interact with the immunoglobulin and ITIM domain (TIGIT) receptor mainly expressed on T cells and NK cells (49). The binding of Fap2 to TIGIT could inhibit the activity of NK cells against the tumor cells, leading to the growth and progression of CRC (17). OmpA has multiple functions in bacterial pathogenesis, including adherence to host cells, and induction of host cell death and serum resistance (50). OmpA proteins from *B. fragilis* were found to induce the release and expression of IL-1alpha, TNF-α, IFN-γ, IL-6, and IL-10 from murine splenocytes (51). Monika et al. found that the interactoin between Cu(II) ions and FomA protein (another OMP) can stimulate colon cells to produce reactive oxygen species (ROS), which can lead to DNA damage and lipid peroxidation (52). These OMP proteins (i.e., Fap2, OmpA, and FomA) appear to contribute to the formation and pathogenesis of the CRC.

**Figure 6:**
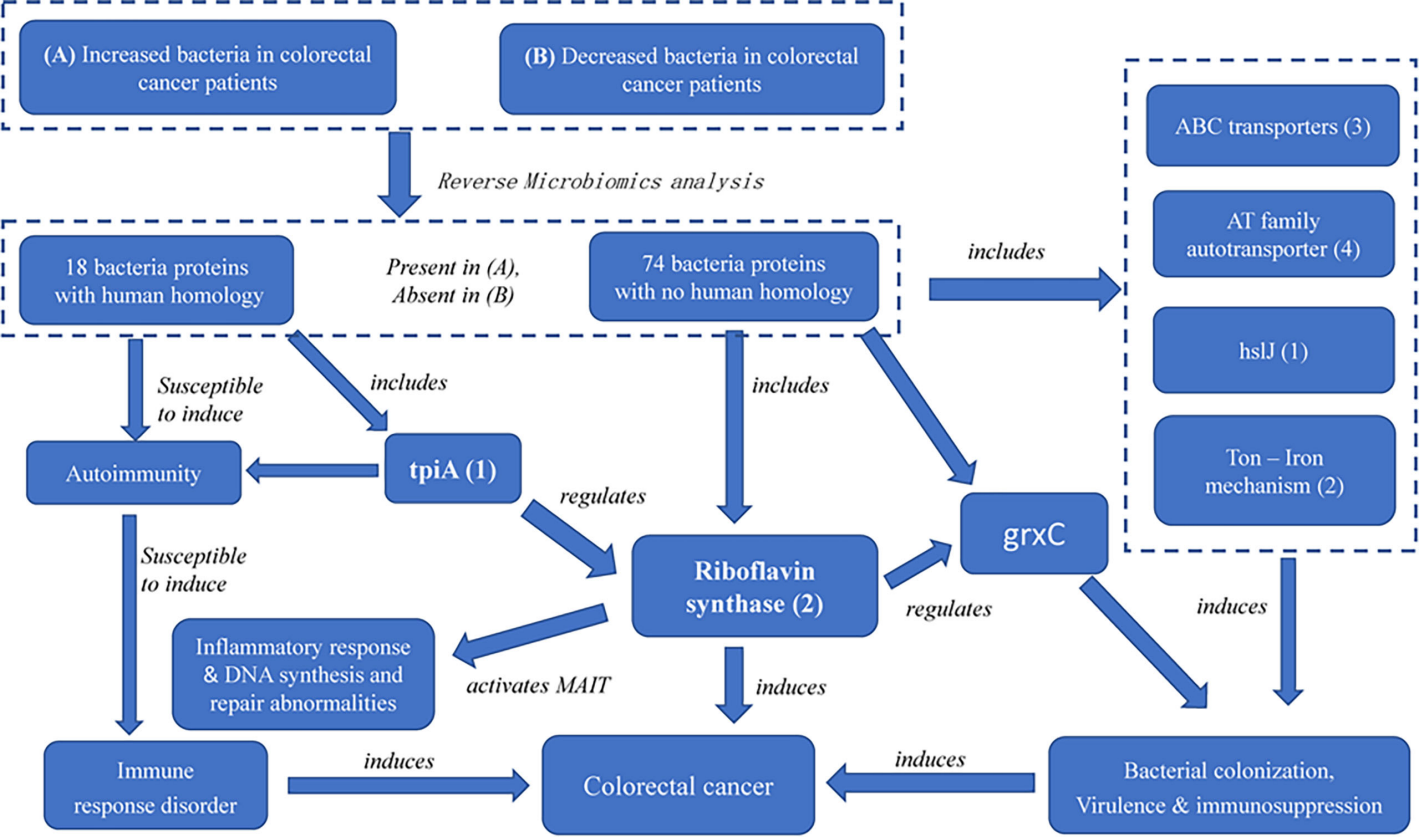
The possible pathogenic mechanism of bacterial-derived riboflavin in CRC. Riboflavin synthase mediates a critical link among ABC transporters proteins, AT family autotransporter, HslJ, Ton, TpiA and GrxC. In the PPI network, TpiA is a upstream regulatory protein, and the GrxC is a downstream protein of riboflavin synthesis. All these proteins, with riboflavin synthesis being a central hub, likely form an interactive PPI network of the CRC formation.

While riboflavin synthase has been regarded as an important CRC risk factor, our study provides the first evidence of two novel discoveries: (i) the riboflavin synthase from enriched bacteria in the colon, which is absent in depleted bacteria in the colon, is likely the virulence factor of CRC, and (ii) we identified its up- and down-stream factors that function together to form a possible pathogenic CRC pathway network. Based on these RM results, we have also generated a model of the CRC pathogenesis (**Figure 6**) with more details described below.

Our identification of riboflavin synthase from enriched but not depleted bacteria in the colons of CRC patients is a signification contribution. The role of riboflavin in CRC has been controversial based on previous epidemiological observational and experimental studies. Some studies indicated that higher intake riboflavin was inversely associated with risk of colorectal cancer. Lack of riboflavin may result in an increase in DNA single-strand breaks by carcinogens, and abnormal DNA repair, eventually leading to CRC (53, 54). In contrast, many studies identified riboflavin as a virulence factor of CRC (55). However, some other studies showed no association between CRC and riboflavin (56). We argue that the riboflavin generated from the microbiota in colon is a pathogenic factor in CRC. Humans are unable to synthesize riboflavin and are therefore dependent on diet and microbe-derived riboflavin. As far as we know, most studies have focused on investigating the association of riboflavin intakes from diet with the risk of CRC (53, 57). The majority of dietary riboflavin is absorbed in small intestine. On the other hand, the bacterial riboflavin is produced and absorbed in the large intestine (58). The existance of different sources of riboflavin may explain the phenomenon that the serum riboflavin concentration is different from the riboflavin concentration in colonic mucosa, and it may also be the reason for the current controversy about the role of riboflavin in CRC. To the best of our knowledge, we are the first to report the important role of riboflavin synthase in the colons to the incidence and development of CRC.

The role of riboflavin synthase in CRC development may be due to the activity of microbe-derived riboflavin metabolites in activating mucosal-associated invariant T-Cell (MAIT) in a MR1-dependent manner (59). Recent researches have demonstrated that MAIT cells involve in a broad range of infectious and non-infectious diseases, including cancers, autoimmunity, allergies and inflammatory disorders (60). The bacterial-derived riboflavin metabolites could activate MAIT cells at the site of colonic mucosa, and produce pro-inflammatory cytokines (including IFN-γ, TNF-a, and IL-17), and express cytotoxic molecules (including granzymes, granulysin and perforin), resulting in chronic inflammatory of the intestinal tract (61). Emerging evidence indicates that more MAIT cells infiltrate CRC tumor tissues than infiltrate healthy colorectal tissues (62, 63). MAIT cells in CRC infiltrates express high levels of the IL-13 receptor, which promotes tumor progression and correlate with a poor prognosis (64, 65).

Furthermore, our study identified many factors at the upstream and downstream of riboflavin synthase. Our pathway analysis indicated that the riboflavin metabolism pathway was the most significant pathway of the PPI network (**Figures 4**, **6**). As described before, many of the hub proteins including riboflavin, ABC transporter proteins, and heat shock protein are closely related to the pathological process of CRC, and the riboflavin synthesis in the colon is likely critical to CRC formation. At the upstream of riboflavin synthase in our identified PPI network, TpiA is a regulatory protein that affects the metabolism, virulence, and antibiotic resistance of bacteria (66). TpiA is also a human autoantigen susceptible to induce autoimmune response. Meanwhile, the downstream protein GrxC is a kind of antioxidant enzyme that may contribute to the survival and metastasis of several types of cancer. In oral squamous cell carcinoma (OSCC), GrxC promotes migration and invasion of tumor cell *via* the Notch signalling pathway (67). He et al. found that GrxC promotes nasopharyngeal carcinoma growth and metastasis through EGFR/Akt pathway (68). Other proteins in the PPI network, such as the heat shock protein HslJ, play an important roles in cancer metabolism. Hsp70 has also been found to mediate tumor cell transformation-progression (69). ABC transporter protein and AT family autotransporter proteins have also been associated with multidrug resistance and transport of materials such as metals and amino acids (37). All these factors, with riboflavin synthesis being a central hub, likely form an interactive PPI network of the CRC formation (**Figure 6**).

One limitation of the current RM study is the lack of experimental verification. However, our RM study provides many valuable hypotheses for further experimental verification. Current RM study predicted many autoantigens, virulence factors, and possible pathogenesis pathways leading to CRC. For example, our study predicts the molecular mechanisms about how the bacterial-derived riboflavin and other pathogenic proteins affect the occurrence and progression of CRC. Those pathogenic proteins factors including riboflavin synthase are likely to be excellent targets in our deep understanding of the CRC mechanism, and rational vaccine and drug design against CRC. These hypotheses deserve further experimental verification.

The existing Reverse Microbiomics (RM) strategy still has limitations and can be further improved. Current work analyzed the genomes/proteomes from 6 enriched and 4 depleted bacterial species, which form a small portion of the total of 146 enriched or depleted bacteria found in our study. While our careful selection of the 10 enriched or depleted species made our RM study focused and feasible, it would be ideal to use all or the majority of the identified bacteria for more systematic study. In addition to the proteome sequence analysis, it would also be good to use other types of data, such as high throughput gene expression omics data or literature mining data, for more powerful RM analysis, which will be investigated later.

In the future, we also plan to generate new algorithms and methods for more advanced RM analyses. The Vaxign tool used in our study was originally designed for reverse vaccinology. While it is suitable for current RM analysis, it would be ideal to develop a RM-specific program(s) or tools for more specific data processing, integration, and analysis of various microbiome datasets or knowledge. The Ontology of Host-Microbiome Interactions (OHMI) (26) can also be further developed and applied for interoperable data/knowledge standardization, annotation, and anaysis, leading to more advanced research of the deep interactions between hosts and microbiomes under different conditions. We also anticipate that the RM strategy will be used by us or others to study more microbiome-related diseases, leading to more productive basic and translational research.

## Conclusions

This study applied the newly developed Reverse Microbiomics (RM) method to systematically study and predict autoantigens and virulence factors that likely contribute to the CRC pathogenicity. Our literature mining found 63 valuable bacterial species up- or down-regulated in the microbiomes of the colons from the CRC patients. The RM strategy was further used to predict 18 autoantigens and 76 potential virulence factors associated with CRC. While many of these autoantigens and virulence factors have been experimentally verified, our study predict many new pathogenetic factors contributing to the CRC genesis. A major finding is the identification of riboflavin synthase and its associated riboflavin metabolism in the colon as a major risk factor of CRC. The riboflavin synthase is present in all up-regulated bacterial species and absent in all down-regulated species in the colorectal microbiota of CRC. Furthermore, many other factors such as TpiA and GrxC are also identified. A new systematic CRC formation model (**Figure 6**) was further proposed to illustrate how riboflavin synthase and other related proteins act to result in the CRC development.

## Data Availability Statement

The datasets presented in this study can be found in online repositories. The names of the repository/repositories and accession number(s) can be found in the article/**Supplementary Material**.

## Author Contributions

HW contributed to literature mining and annotation, ontology representation, RM, implementation, data analysis, result interpretation, and project design. KZ contributed to CRC use case knowledge extraction and data analysis. LW contributed to data analysis. QQ contributed to CRC use case knowledge extraction and result interpretation. YH contributed to project design, ontology development, and data analysis. HW and YH prepared the first draft of the article. All authors read, edited, and agreed on the article submission and publication.

## Funding

This work was supported by a grant to HW from the Natural Science Foundation of Heilongjiang Province (Grant No. LH2019C074) and a grant to YH from the Michigan Medicine–Peking University Health Sciences Center Joint Institute for Clinical and Translational Research (U063430).

## Conflict of Interest

The authors declare that the research was conducted in the absence of any commercial or financial relationships that could be construed as a potential conflict of interest.

## Publisher’s Note

All claims expressed in this article are solely those of the authors and do not necessarily represent those of their affiliated organizations, or those of the publisher, the editors and the reviewers. Any product that may be evaluated in this article, or claim that may be made by its manufacturer, is not guaranteed or endorsed by the publisher.
