# Supplementary material for: Prediction of Pathogenic Factors in Dysbiotic Gut Microbiomes of Colorectal Cancer Patients Using Reverse Microbiomics
Source: Front Oncol. 2022 Apr 27;12:882874. doi: 10.3389/fonc.2022.882874 (PMC9091335; doi:10.3389/fonc.2022.882874)
Supplement: Supplementary file 3 [file Table_3.docx]

|  |
| --- |

**Supplementary Table 3 47 complete gut microbiome genomes used in the RM study**

| NO. | **Species** | **Strain** | **NCBI Bio Project No.** | **Proteins number** | **Alterations** |
| --- | --- | --- | --- | --- | --- |
| **Group:** ***Bacteroides*** | | | | | |
| 1 | *B. fragilis* | YCH46 | PRJNA13067 | 4278 | Up-regulation |
| 2 | *B. fragilis* | 638R | PRJNA50405 | 4193 | Up-regulation |
| 3 | *B. fragilis* | BOB25 | PRJNA278510 | 4137 | Up-regulation |
| 4 | *B. fragilis* | S14 | PRJNA290855 | 4059 | Up-regulation |
| 5 | *B. ovatus* | ATCC 8483 | PRJNA289334 | 4751 | Up-regulation |
| 6 | *B. uniformis* | An67 | PRJNA377666 | 3595 | Down-regulation |
| 7 | *B. uniformis* | ATCC 8492 | PRJNA18195 | 3701 | Down-regulation |
| 8 | *B. uniformis* | CL03T00C23 | PRJNA64833 | 4019 | Down-regulation |
| 9 | *B. uniformis* | dnLKV2 | PRJNA175976 | 3833 | Down-regulation |
| 10 | *B. uniformis* | KLE1607 | PRJNA243490 | 3775 | Down-regulation |
| 11 | *B. uniformis* | 3978 T3 ii | PRJNA206129 | 3917 | Down-regulation |
| 12 | *B. uniformis* | CL03T12C37 | PRJNA64835 | 3980 | Down-regulation |
| 13 | *B. finegoldii* | CL09T03C10 | PRJNA64831 | 4010 | Down-regulation |
| 14 | *B. finegoldii* | DSM 17565 | PRJNA27823 | 3773 | Down-regulation |
| 15 | *B. intestinalis* | DSM 17393 | PRJNA20523 | 4494 | Down-regulation |
| 16 | *B. intestinalis* | KLE1704 | PRJNA257717 | 5037 | Down-regulation |
| **Group:** ***Fusobacterium*** | | | | | |
| 17 | *F. nucleatum* | ATCC 10953 | PRJNA17261 | 2238 | Up-regulation |
| 18 | *F. nucleatum* | 7_1 | PRJNA32483 | 2335 | Up-regulation |
| 19 | *F. nucleatum* | KCOM 1279 | PRJNA270143 | 2239 | Up-regulation |
| 20 | *F. nucleatum* | ATCC 25586 | PRJNA295 | 2067 | Up-regulation |
| 21 | *F. nucleatum* | 4_8 | PRJNA32481 | 2020 | Up-regulation |
| 22 | *F. nucleatum* | ChDC F306 | PRJNA270146 | 2391 | Up-regulation |
| 23 | *F. nucleatum* | 3_1_27 | PRJNA41563 | 1913 | Up-regulation |
| 24 | *F. nucleatum* | 3_1_36A2 | PRJNA38357 | 2063 | Up-regulation |
| 25 | *F. periodonticum* | 2_1_31 | PRJNA32473 | 2276 | Up-regulation |
| 26 | *F. periodonticum* | ATCC 33693 | PRJNA30495 | 2352 | Up-regulation |
| 27 | *F. periodonticum* | D10 | PRJNA32469 | 2189 | Up-regulation |
| **Group:** ***Streptococcus*** | | | | | |
| 28 | *S. equinus* | ATCC 700338 | PRJNA50519 | 1862 | Up-regulation |
| 29 | *S. equinus* | ATCC 9812 | PRJNA52999 | 1544 | Up-regulation |
| 30 | *S. equinus* | HC5 | PRJNA245446 | 1714 | Up-regulation |
| 31 | *S. equinus* | FDAARGOS_251 | PRJNA231221 | 2050 | Up-regulation |
| 32 | *S. gallolyticus* | DD02 | PRJNA304333 | 2100 | Up-regulation |
| 33 | *S. gallolyticus* | DSM 16831 | PRJNA357355 | 2356 | Up-regulation |
| 34 | *S. gallolyticus* | NTS 31307655 | PRJNA316443 | 2226 | Up-regulation |
| 35 | *S. gallolyticus* | NTS31301958 | PRJNA327032 | 2231 | Up-regulation |
| 36 | *S. gallolyticus* | TX20005 | PRJNA30645 | 2125 | Up-regulation |
| 37 | *S. thermophilus* | LMD-9 | PRJNA13773 | 1645 | Down-regulation |
| 38 | *S. thermophilus* | ASCC 1275 | PRJNA222865 | 1625 | Down-regulation |
| 39 | *S. thermophilus* | CNRZ1066 | PRJNA13163 | 1591 | Down-regulation |
| 40 | *S. thermophilus* | CS8 | PRJNA326481 | 1593 | Down-regulation |
| 41 | *S. thermophilus* | LMG 18311 | PRJNA13162 | 1564 | Down-regulation |
| 42 | *S. thermophilus* | M17PTZA496 | PRJNA230629 | 1851 | Down-regulation |
| 43 | *S.thermophilus* | MN-ZLW-002 | PRJNA159887 | 1671 | Down-regulation |
| 44 | *S. thermophilus* | ND03 | PRJNA49149 | 1667 | Down-regulation |
| 45 | *S. thermophilus* | S9 | PRJNA307645 | 1603 | Down-regulation |
| 46 | *S. thermophilus* | SMQ-301 | PRJNA251645 | 1660 | Down-regulation |
| 47 | *S. thermophilus* | TH982 | PRJNA231305 | 1602 | Down-regulation |
